# Neutrophil-to-Lymphocyte Ratio, Platelet-to-Lymphocyte Ratio and Heart Failure

**DOI:** 10.5935/abc.20160037

**Published:** 2016-03

**Authors:** Viroj Wiwanitkit

**Affiliations:** Hainan Medical University, China

**Keywords:** Neutrophils, Heart Failure, Lymphocytes

Dear Editor, the recent report on “Neutrophil-to-Lymphocyte Ratio (NLR), Platelet-to-Lymphocyte Ratio (PLR) and Heart Failure” is very interesting.^1^ Durmus et al.^1^ reported that “NLR can be used to predict mortality during the follow-up of HF patients.” Indeed, the use of a new parameter for predicting heart failure is very interesting. However, as Durmus et al.^1^ mentioned, both NLR and PLR “were not sufficient to establish a diagnosis of HF.” In addition, although the present report^1^ was well designed as a matched case-control study, the problem on NLR and PLR should be discussed in view of laboratory medicine. Both parameters are non - specific. Several factors can affect neutrophils, lymphocytes and platelets. Other concomitant disorders such as immunological and malignant disorders can alter the NLR and PLR values.^2-3^ Also, metabolic syndrome, which is common among the patients with cardiac disease, can also affect NLP and PLR values.^4^ In addition, different automated hematological analyzers can also give different results of neutrophil, lymphocyte and platelet measurements.^5^
