# Supplementary material for: Neutrophil-to-Lymphocyte Ratio, Platelet-to-Lymphocyte Ratio and Heart Failure
Source: Arq Bras Cardiol. 2016 Mar;106(3):265–6. doi: 10.5935/abc.20160037 (PMC4811284; doi:10.5935/abc.20160037)
Supplement: Related texts

# Reply

**Authors:** Erdal Durmus, Tarik Kivrak, Fethullah Gerin, Murat Sunbul, Ibrahim Sari, Okan Erdogan

We recently published a trial named "Neutrophil-to-Lymphocyte Ratio (NLR) and Platelet-to-Lymphocyte Ratio (PLR) are Predictors of Heart Failure (HF)".^1^ In this study, we found that NLR can be used to predict mortality during the follow-up of HF patients. However, NLR and PLR were not sufficient to establish a diagnosis of HF. According to guidelines, HF diagnosis is generally made based on clinical signs and symptoms of patients. Other examinations and laboratory test are used to support or to rule out the HF diagnosis.^2^ Although NLR and PLR were higher in HF patients when compared to healthy controls, they are not sufficient, alone, to establish the diagnosis of HF. Use of n-terminal pro brain natriuretic peptide, a commonly used biomarker in congestive HF management, alone, is also not enough for diagnosis of HF, as the use of NLR alone.^3^ Another problem regarding the use of NLR and PLR in HF patients is the lack of specificity. NLR and PLR are ratios of different subgroups of blood cells. So, they should be affected in various disease situations. Neutrophil count may be increased in patients with a bacterial infection and platelets count may also be increased in patients with essential thrombocythemia. In our study, to minimize this problem, we excluded patients who had malignancies, connective tissue diseases, acute and chronic infections, renal disease and acute coronary syndromes.

As mentioned in the letter, NLR and PLR can be affected by metabolic syndrome. In our study population, we did not assess the presence of metabolic syndrome. However, there was no difference between the NLR and PLR values of patients with or without hypertension (HT), hyperlipidemia (HL) and diabetes mellitus (DM).^4^ In a previously reported study, NLR levels tended to be high in patients with HT and DM. Finally, another criticism is that "different automated hematological analyzers can also give different results of neutrophil, lymphocyte and platelet measurements". This is true, because different automated hematological analyzers use different techniques.^5^ But, considering the basic blood cell count, there was a good correlation between different automated hematological analyzers.^6^ And also, NLR and PLR are ratios of blood cells parameters, so they are not affected by the type of analyzers.

## References

[1] Durmus E, Kivrak T, Gerin F, Sunbul M, Sari I, Erdogan O (2015). Neutrophil-to-lymphocyte ratio and platelet-to-lymphocyte ratio are
predictors of heart failure. Arq Bras Cardiol.

[2] McMurray JJ, Adamopoulos S, Anker SD, Auricchio A, Böhm M, Dickstein K, ESC Committee for Practice Guidelines (2012). ESC Guidelines for the diagnosis and treatment of acute and chronic
heart failure 2012: The Task Force for the Diagnosis and Treatment of Acute and
Chronic Heart Failure 2012 of the European Society of Cardiology. Developed in
collaboration with the Heart Failure Association (HFA) of the
ESC. Eur Heart J.

[3] Januzzi JL, van Kimmenade R, Lainchbury J, Bayes-Genis A, Ordonez-Llanos J, Santalo-Bel M (2006). NT-proBNP testing for diagnosis and short-term prognosis in acute
destabilized heart failure: an international pooled analysis of 1256 patients:
the International Collaborative of NT-proBNP Study. Eur Heart J.

[4] Imtiaz F, Shafique K, Mirza SS, Ayoob Z, Vart P, Rao S (2012). Neutrophil lymphocyte ratio as a measure of systemic inflammation
in prevalent chronic diseases in Asian population. Int Arch Med.

[5] McNair E, Qureshi AM, Bally C (2015). Performance evaluation of the Plateletworks® in the
measurement of blood cell counts as compared to the Beckman Coulter Unicel DXH
800. J Extra Corpor Technol.

[6] Bruegel M, Nagel D, Funk M, Fuhrmann P, Zander J, Teupser D (2015). Comparison of five automated hematology analyzers in a university
hospital setting: Abbott Cell-Dyn Sapphire, Beckman Coulter DxH 800, Siemens
Advia 2120i, Sysmex XE-5000, and Sysmex XN-2000. Clin Chem Lab Med.
